# Author Correction: The SARS-CoV-2 spike protein is vulnerable to moderate electric fields

**DOI:** 10.1038/s41467-022-31424-y

**Published:** 2022-06-29

**Authors:** Claudia R. Arbeitman, Pablo Rojas, Pedro Ojeda-May, Martin E. Garcia

**Affiliations:** 1 Theoretical Physics and Center for Interdisciplinary Nanostructure Science and Technology, FB10, Universität Kassel, Kassel, Germany; 2 CONICET Consejo Nacional de Investigaciones Científicas y Técnicas, Buenos Aires, Argentina; 3 GIBIO-Universidad Tecnológica Nacional-Facultad Regional Buenos Aires, Buenos Aires, Argentina; 4 High Performance Computing Center North (HPC2N), Umeå University, Umeå, Sweden

**Keywords:** Computational biophysics, Physical sciences

Correction to: *Nature Communications* 10.1038/s41467-021-25478-7, published online 13 September 2021.

The original version of this Article contained two instances of an error in the methods section, which incorrectly read ‘…using the CHARMM software (v. 43a1)’. The correct version states ‘(v. 45b1)’ in place of ‘(v. 43a1)’. This has been corrected in both the PDF and HTML versions of the Article.

