# Supplementary material for: The SARS-CoV-2 spike protein is vulnerable to moderate electric fields
Source: Nat Commun. 2021 Sep 13;12:5407. doi: 10.1038/s41467-021-25478-7 (PMC8437970; doi:10.1038/s41467-021-25478-7)
Supplement: Supplementary file 1 — Supplementary Information [file 41467_2021_25478_MOESM1_ESM.pdf]

Supplementary Information

The SARS-CoV-2 Spike Protein is vulnerable to moderate electric fields

Claudia R. Arbeitman, Pablo Rojas, Pedro Ojeda-May & Martin E. Garcia

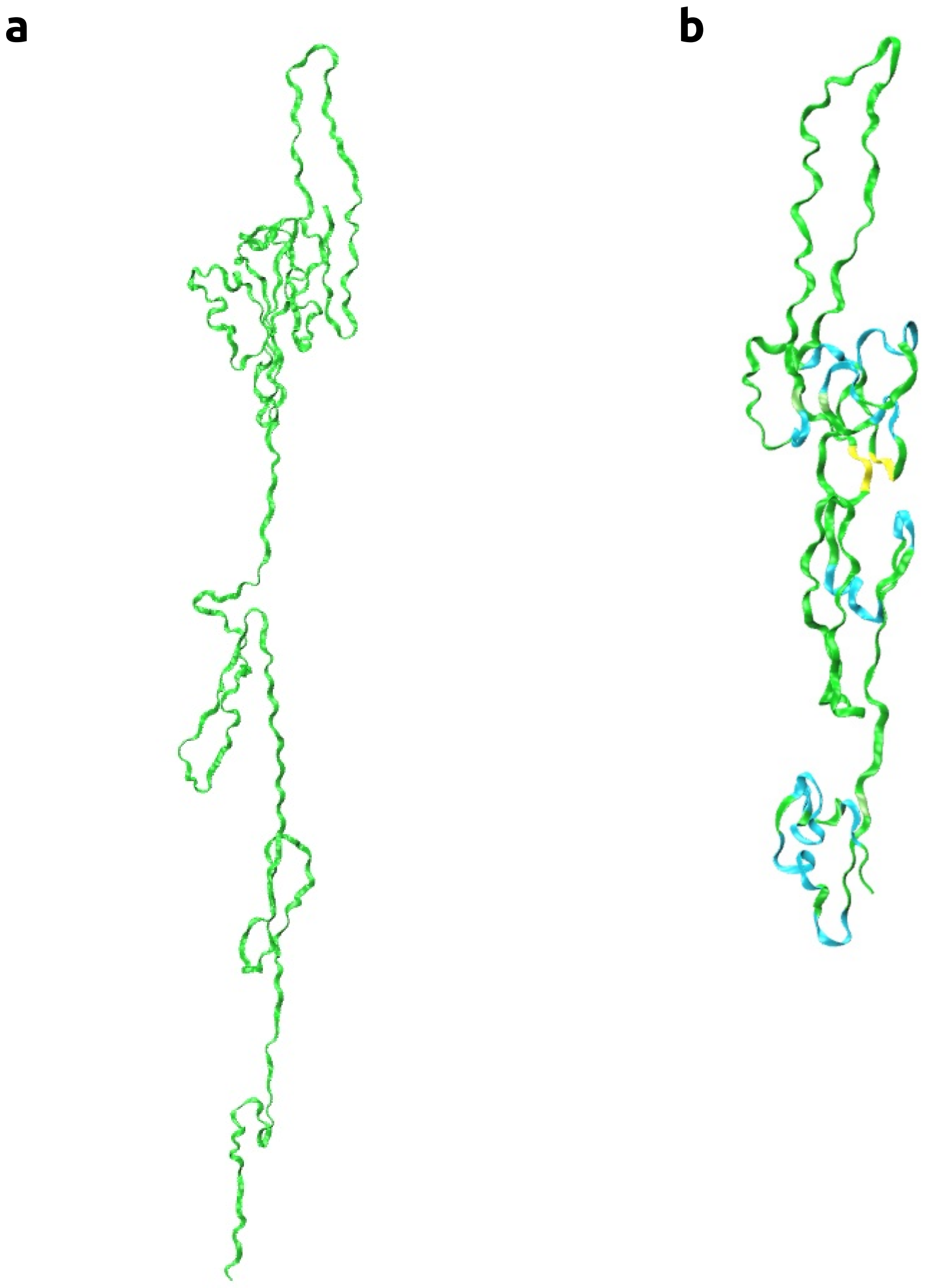

Supplementary Figure 1. Snapshots of **a.** the studied fragment of S PDB ID: 6VSB and **b.** RBD PDB ID: 6M0J under an extreme and experimentally not very relevant EF of 10^{9} V m^{-1}, shown as a limiting case for the sake of comparison. Severe denaturation with complete loss of the secondary and tertiary structures of the protein within the first 1 ns takes place.

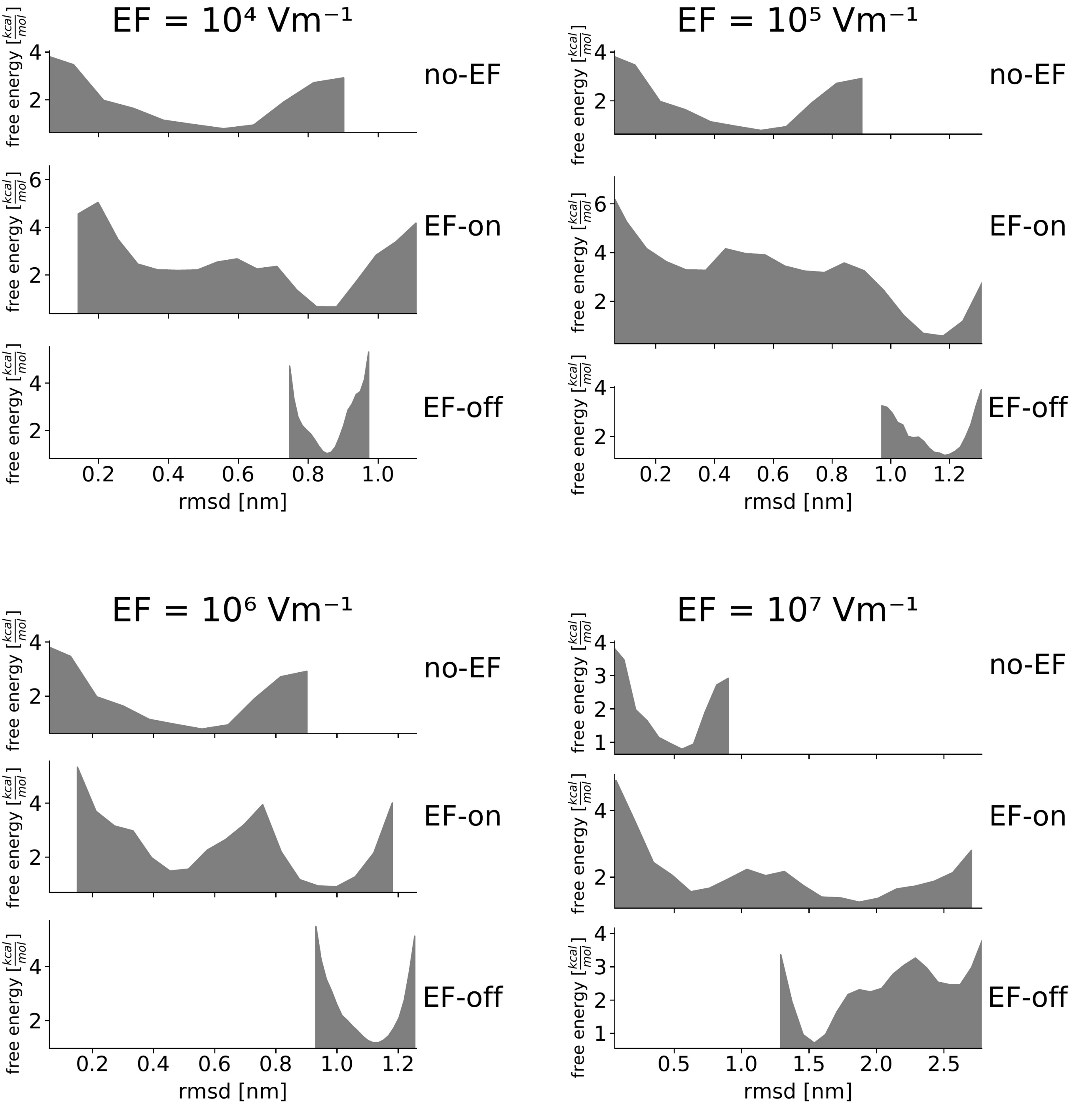

Supplementary Figure 2. Estimated free energy profiles for no EF (top), EF-on (middle) and EF-off (bottom) corresponding to 10^{4} V m^{-1}, 10^{5} V m^{-1}, 10^{6} V m^{-1}, 10^{7} V m^{-1}. For all the EF strengths, the same qualitative landscape is observed, in which the protein state evolves until a new stable minimum is found and its basin is able to confine its further evolution. They also show that an energy barrier prevents a transition back to original conformation.

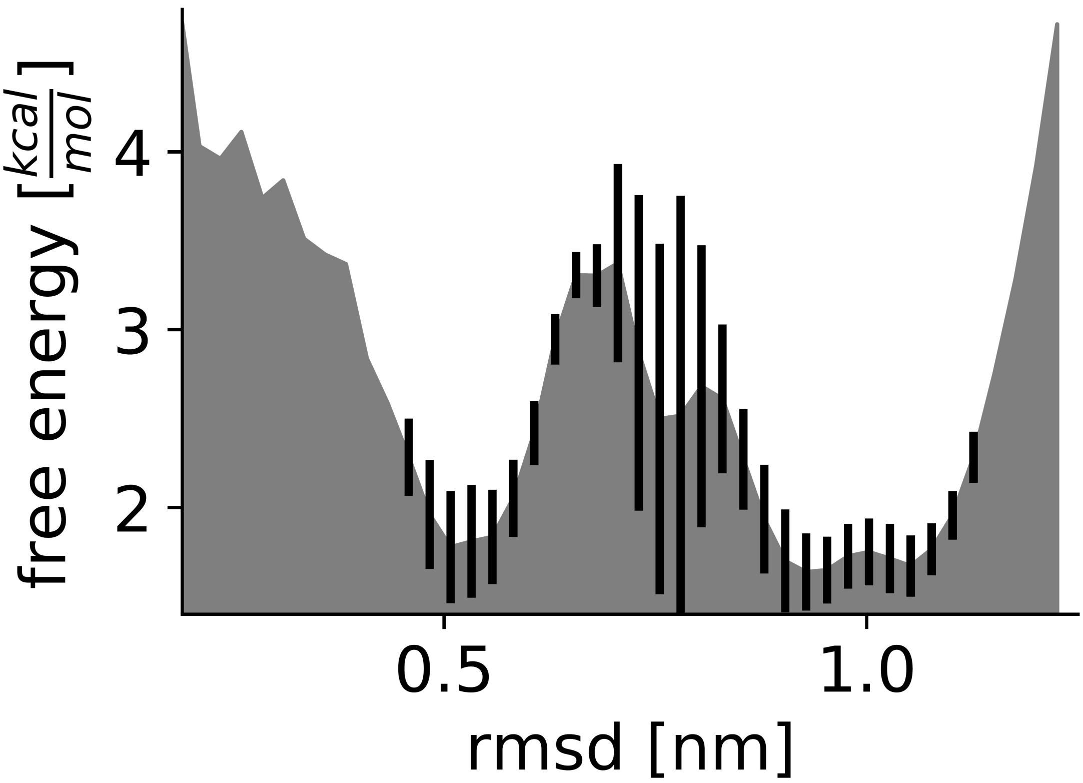

Supplementary Figure 3. Estimate of the free energy profile (EF 10^{6} V m^{-1}) combining the off-field (EF-off) and the field induced configurations in absence of electric fields. The error bars were obtained by means of bootstrapping with replacement (see Methods), and represent the standard deviation of the 5000 re-computed energy profiles. Bars are centred in the values without bootstrapping. For clarity, error bars are not drawn in the extremes. Source Data is provided with this paper.

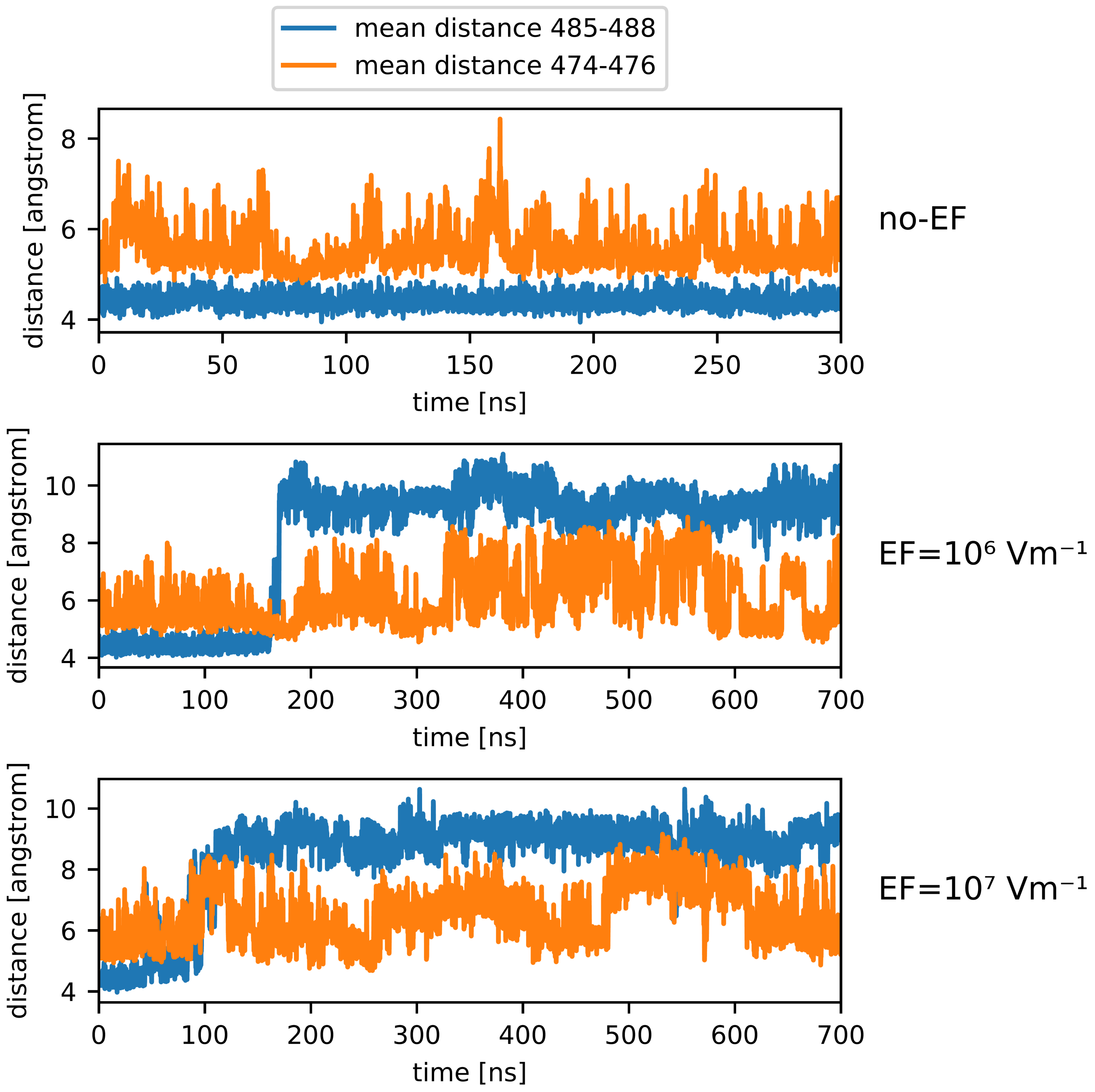

Supplementary Figure 4. Time evolution of the mean distance between residues 485-488 and 474-476 under EFs for different strengths (no-EF and EF-on runs). Application of an external electric field has a significant effect on residues distance. In no-EF simulations, the corresponding distances between residues were observed to remain within values around 4-5 Å that enable these interactions. During EF-on, the same distances increase up to values between 8 Å and 10 Å, which causes a weakening of the inter-residue interaction.

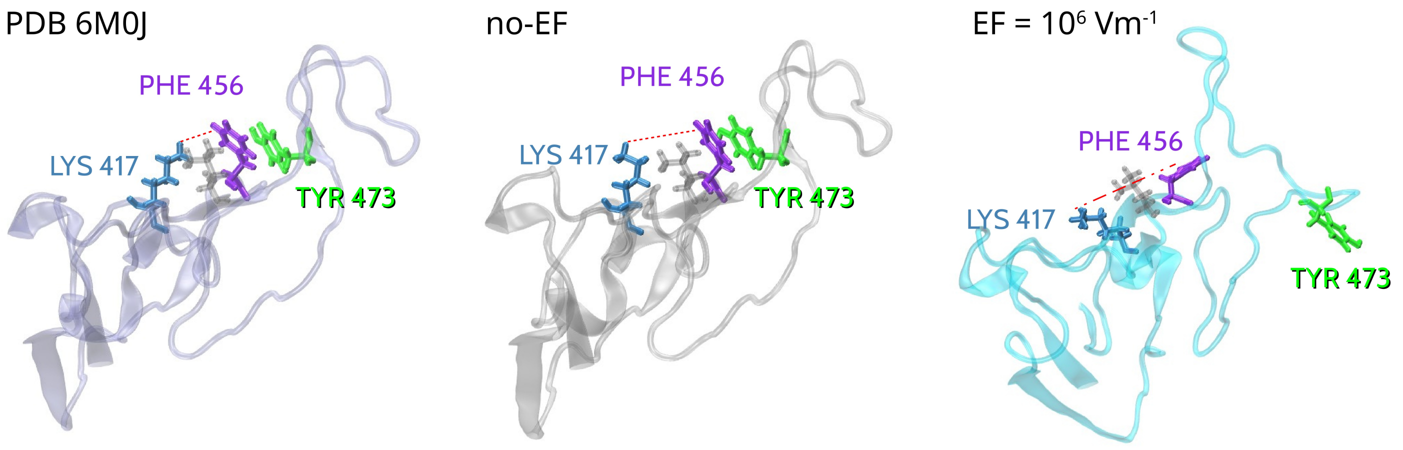

Supplementary Figure 5. Snapshots of the RBM region for PDB ID 6M0J, EF=0 and EF=10^{6} V m^{-1} showing the contact interaction Phe456-Tyr473 and π-cation interaction between residue Phe456 and the side chain of Lys417. Application of an EF increased mobility of L3 leads to a break of these Phe456 close-contact interactions.

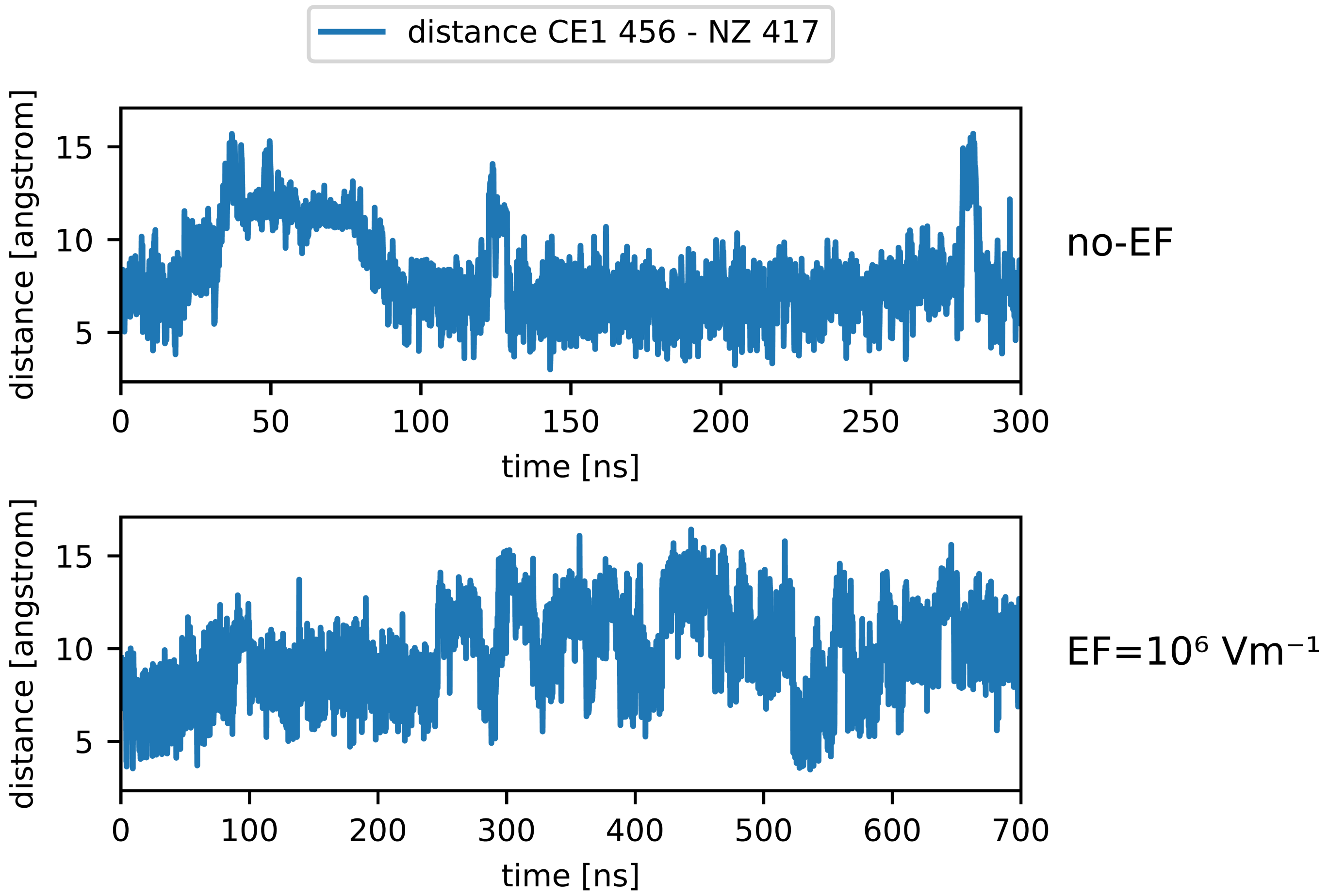

Supplementary Figure 6. Time evolution of the mean distance between residues 417-456 under no-EF and EF-on (10^6 V m^{-1}) runs.

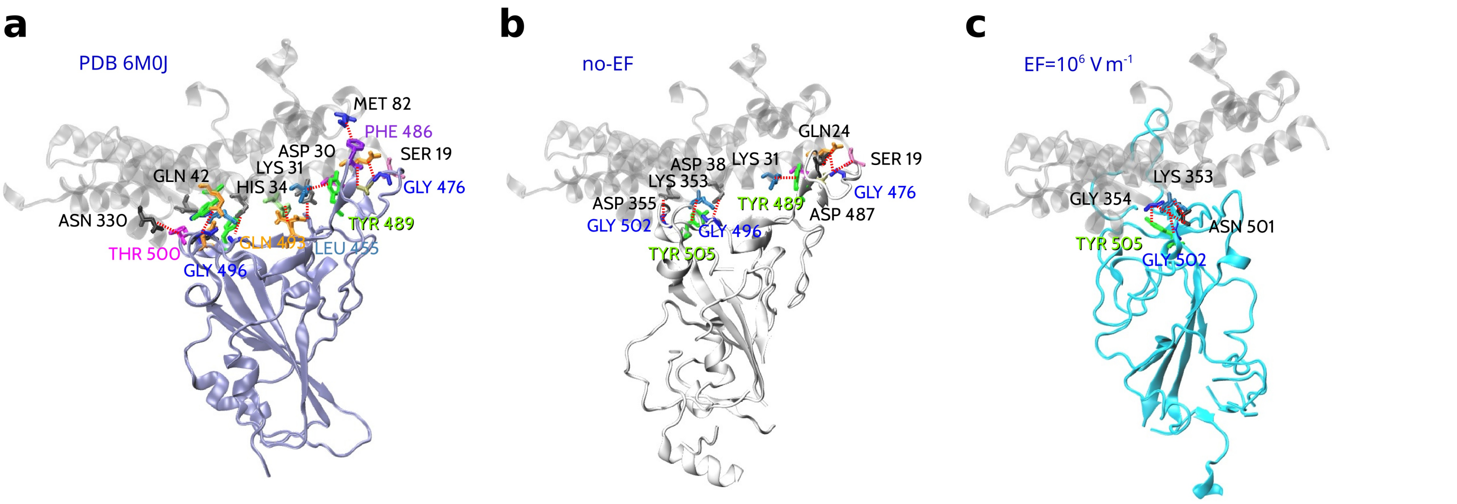

d

Native contacts		PDB ID 6M0J						no-EF						EF=10^6 Vm^{-1}						EF=10^7 Vm^{-1}					
ACE2	RBD	1	2	3	4	5	6	1	2	3	4	5	6	1	2	3	4	5	6	1	2	3	4	5	6
Ser19	Gly476							■	■		■														
Gln24	Ala475	■	■	■	■			■	■		■														
Gln24	Gly476	■	■	■				■	■		■														
Gln24	Asn487	■			■			■	■	■									■						
Tyr27	Ala475	■	■					■	■		■													■	
Asp30	Leu455	■	■	■			■		■		■						■	■		■					
Lys31	Tyr489	■	■	■	■		■	■		■										■	■			■	
His34	Gln493			■	■		■			■										■	■				■
Asp38	Gly496	■						■																	■
Tyr41	Gln498	■	■										■		■										
Gln42	Gly446	■	■	■						■			■												
Met82	Phe486	■	■	■	■					■															
Asn330	Thr500	■	■														■						■		
Lys353	Asn501	■	■		■	■						■		■	■							■			
Lys353	Gly502	■	■						■			■		■	■								■		
Lys353	Tyr505	■	■					■	■		■				■										
Gly354	Asn501	■	■	■	■	■				■		■	■	■						■		■			
Gly354	Gly502	■	■													■				■	■	■			
Gly354	Tyr505	■	■			■								■		■					■				
Asp355	Thr500	■	■	■	■					■	■		■							■	■				
Asp355	Asn501	■	■			■						■													
Asp355	Gly502	■	■			■		■	■	■															

Supplementary Figure 7. Snapshots showing RBD-ACE2 binding interface. **a.** Native contacts: the 22 contacts (as pairs of residues) between the RBD and ACE2 taken into consideration were extracted from the PDB ID 6M0J crystal structure of the RBD-ACE2 complex (cutoff of 7 Å). **b.,c**. Structures that preserve the highest number of the native contacts for EF= 0 (9/22) and EF= 10^{6} V m^{-1} (4/22). The orientation of the ACE2 binding interface is the same in all figures. **d.** Preserved contacts for the best 6 docking cases. Coloured cells represent contacts that are preserved. Native contacts involving residues from L3 in RBD (Tyr470- Pro491) are particularly affected when EF is applied.

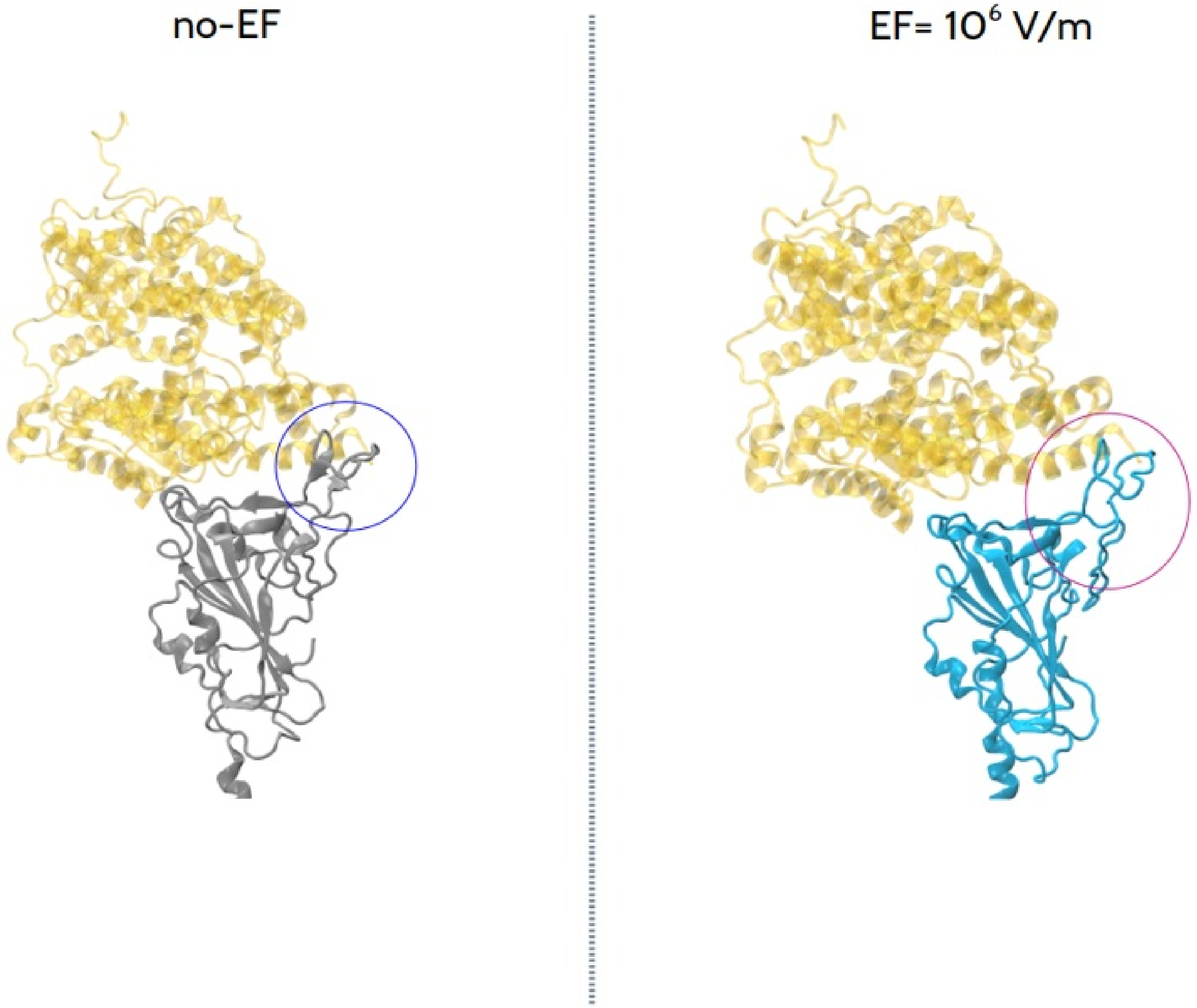

Supplementary Figure 8. EF induces damage on the RBD even under confinement inside the RBD-ACE2 complex. We considered the bound RBD-ACE2 complex and applied an external electric field only to the RBD. This was achieved by implementing position restraints on the back bone atoms and on the heavy atoms of the side chains of ACE2, using large force constant values of 100000.0 kJ mol^{-1} nm^{-2} and 10000.0 kJ mol^{-1} nm^{-2}, respectively. In this way, the EF only affects the RBD in the bound state. Initial simulations with no-EF were performed during 300 ns, and then an EF of strength 10^{6} V m^{-1} was applied during 500 ns. Results of those constrained simulations confirm our previous conclusions on the isolated RBD, namely, that the main EF induced damage occurs in the loop 3 (L3) region before and after application of an EF of 10^{6} V m^{-1} (only on the RBD). The figure shows the full view of the secondary structures of the complex before and after EF application. The orientation of the ACE2 receptor is the same in both figures. The L3 loop in each of the structures is highlighted with a circle. The disappearance of the two β-sheets in the L3 loop of the RBM and a partial RBD-ACE2 dissociation can be clearly observed on the right panel. In comparison with no-EF, the RBM is not as tightly associated after application of an EF.

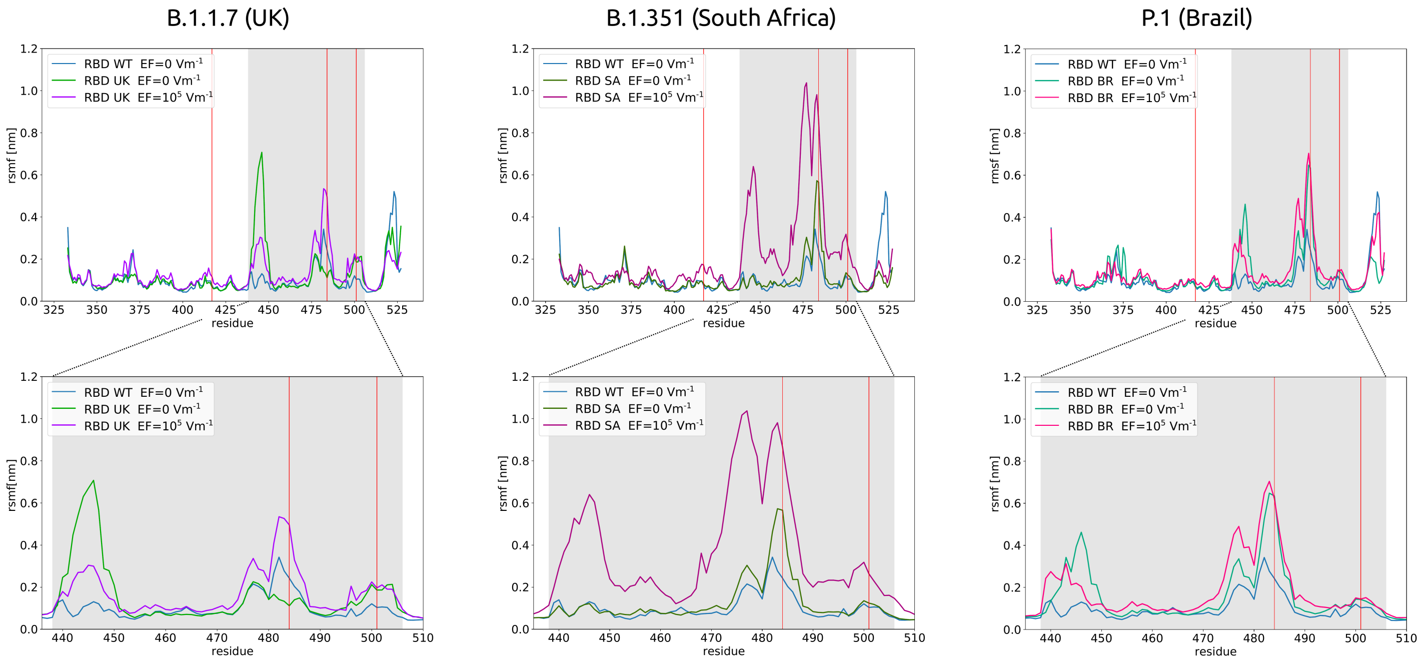

Supplementary Figure 9. Structural flexibility of the RBD of the variants of concern B.1.1.7, B.1.351 and P.1 under EF. Root mean square fluctuations (RMSF) of the RBM region in the VOC in absence and presence of an external field of strength 10 ^{5} V m^{-1} was applied. Even in absence of fields the RBM region shows an increased flexibility in the three variants when compared to wild type. Residues of the RBD that considerably increase their flexibility under EF application, particularly S477, E484 and K484, are critical and situated at the interface region to the ACE2 receptor.

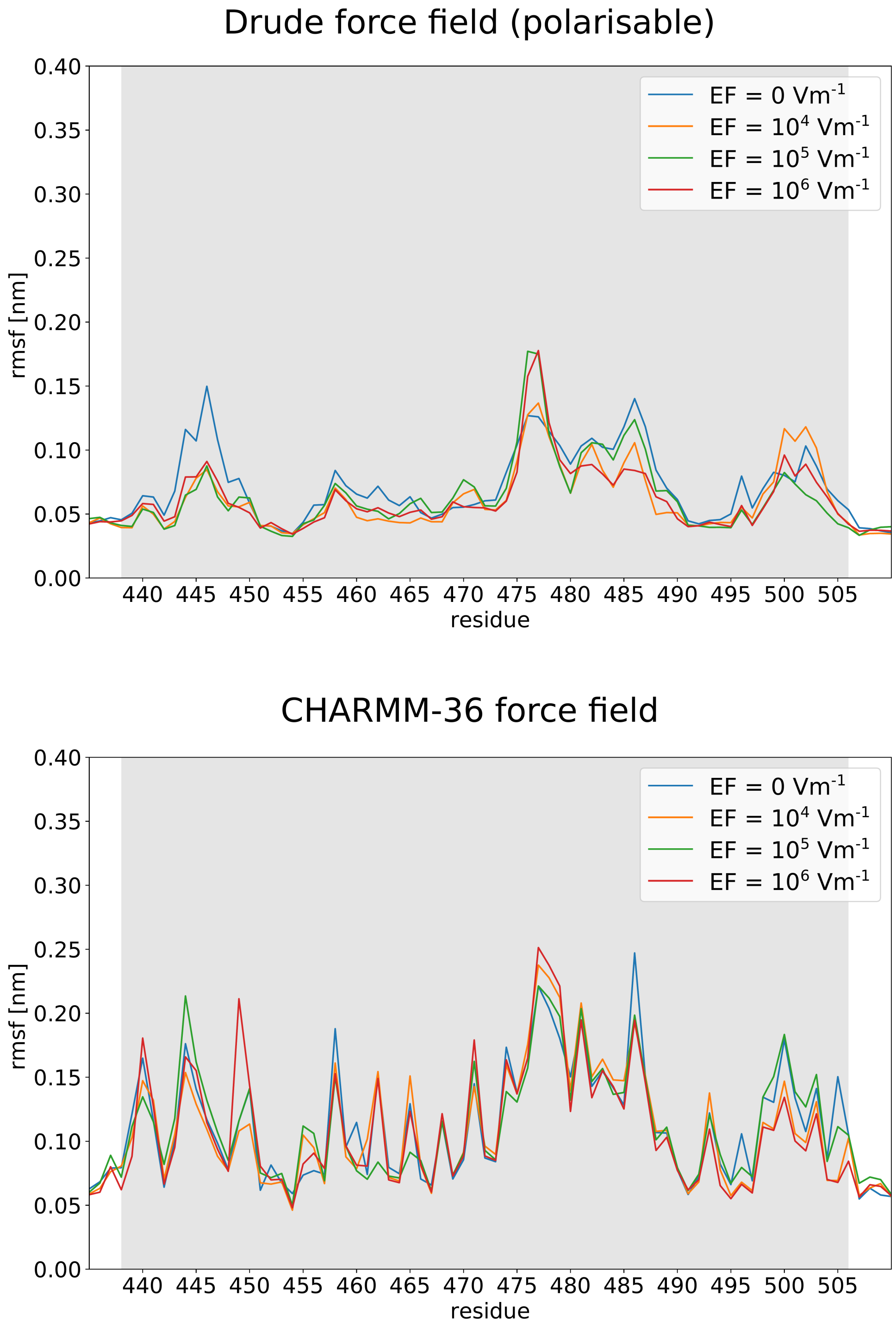

Supplementary Figure 10. Root mean square fluctuations of the RBM (wild type) for different EF strengths using a polarisable and a non-polarisable force field. Upper panel: Drude polarisable force field (simulation over 0.2ns). Lower panel: CHARMM-36 force field (simulation over 1ns).
