# Supplementary material for: The SARS-CoV-2 spike protein is vulnerable to moderate electric fields
Source: Nat Commun. 2021 Sep 13;12:5407. doi: 10.1038/s41467-021-25478-7 (PMC8437970; doi:10.1038/s41467-021-25478-7)
Supplement: Supplementary file 3 — Description of Additional Supplementary Files [file 41467_2021_25478_MOESM3_ESM.pdf]

Description of Additional Supplementary Files

Supplementary Data 1. Parameters and scripts used in the MD simulation and in the analysis tools. Analyses were performed using python >= 3.7 , mdanalysis >= 1.0.0 ,seaborn >= 0.11.0, scikit-learn >= 0.23.2, scipy >= 1.5.2. MD simulation parameters refer to those used in GROMACS 2019.4. Details are provided in the Methods section of the manuscript.
