# Supplementary material for: The SARS-CoV-2 spike protein is vulnerable to moderate electric fields
Source: Nat Commun. 2021 Sep 13;12:5407. doi: 10.1038/s41467-021-25478-7 (PMC8437970; doi:10.1038/s41467-021-25478-7)
Supplement: Supplementary file 5 — Reporting summary [file 41467_2021_25478_MOESM5_ESM.pdf]

nature research

Corresponding author(s): Prof. Martin E. Garcia

Last updated by author(s): Aug 4, 2021

Reporting Summary

Nature Research wishes to improve the reproducibility of the work that we publish. This form provides structure for consistency and transparency in reporting. For further information on Nature Research policies, see our Editorial Policies and the Editorial Policy Checklist.

Statistics

For all statistical analyses, confirm that the following items are present in the figure legend, table legend, main text, or Methods section.

n/a	Confirmed	
[ ]	[x]	The exact sample size (n) for each experimental group/condition, given as a discrete number and unit of measurement
[x]	[ ]	A statement on whether measurements were taken from distinct samples or whether the same sample was measured repeatedly
[x]	[ ]	The statistical test(s) used AND whether they are one- or two-sided<br>*Only common tests should be described solely by name; describe more complex techniques in the Methods section.*
[x]	[ ]	A description of all covariates tested
[x]	[ ]	A description of any assumptions or corrections, such as tests of normality and adjustment for multiple comparisons
[ ]	[x]	A full description of the statistical parameters including central tendency (e.g. means) or other basic estimates (e.g. regression coefficient) AND variation (e.g. standard deviation) or associated estimates of uncertainty (e.g. confidence intervals)
[x]	[ ]	For null hypothesis testing, the test statistic (e.g. F, t, r) with confidence intervals, effect sizes, degrees of freedom and P value noted<br>*Give P values as exact values whenever suitable.*
[x]	[ ]	For Bayesian analysis, information on the choice of priors and Markov chain Monte Carlo settings
[x]	[ ]	For hierarchical and complex designs, identification of the appropriate level for tests and full reporting of outcomes
[x]	[ ]	Estimates of effect sizes (e.g. Cohen's d, Pearson's r), indicating how they were calculated

Our web collection on statistics for biologists contains articles on many of the points above.

Software and code

Policy information about availability of computer code

Data collection	Software: Modeller (v. 9.23), CHARMM software (v. 43a1) with the CHARMM36 force field, CHARMM-GUI, GROMACS (v. 2019.4), NAMD (v. 2.14)<br>Algorithms: LINCS algorithm, PME method, Parrinello-Rahman
Data analysis	GROMACS tools (v. 2019.4), MDAnalysis Python library (v. 1.0.0), Scikit-learn Python library (v. 0.23.0), VMD software package version 1.9.4a38 (2019), STRIDE algorithm included in VMD v. 1.9.4a38 (2019), Adaptive Poisson-Boltzmann Solver (APBS) and PDB2PQR v. 3.0 web server, pyDock v. 3.5.2 web server.

For manuscripts utilizing custom algorithms or software that are central to the research but not yet described in published literature, software must be made available to editors and reviewers. We strongly encourage code deposition in a community repository (e.g. GitHub). See the Nature Research guidelines for submitting code & software for further information.

Data

Policy information about availability of data

All manuscripts must include a data availability statement. This statement should provide the following information, where applicable:

- Accession codes, unique identifiers, or web links for publicly available datasets
- A list of figures that have associated raw data
- A description of any restrictions on data availability

The structural data used in this study were obtained from the RCSB Protein Data Bank (https://www.rcsb.org/) under the PDB IDs 6VSB and 6M0J.
The complete raw simulation data that supports the findings of this study are available upon reasonable request.

Field-specific reporting

Please select the one below that is the best fit for your research. If you are not sure, read the appropriate sections before making your selection.

☒ Life sciences ☐ Behavioural & social sciences ☐ Ecological, evolutionary & environmental sciences

For a reference copy of the document with all sections, see nature.com/documents/nr-reporting-summary-flat.pdf

Life sciences study design

All studies must disclose on these points even when the disclosure is negative.

Sample size	Computational simulations were performed starting from two different independent structures (with PDB IDs 6M0J and 6VSB). For each of the structures, simulations were performed with zero-field (replicated) and different magnitudes of electric field (4 magnitudes for 6VSB and 3 magnitudes for 6M0J, plus an extra case of very high magnitude upon each structure). Since different cases with different structures gave consistent results, we counted them as replications.
Data exclusions	The first 40ns of simulation were ignored to account for an initial stabilisation of the system. In the RMSD analysis, we used a standard procedure where the tails of the protein which fluctuated considerably were not included in the analysis.
Replication	Two different sets of simulations with the PDB structures 6M0J and 6VSB were computed and displayed consistent results.
Randomization	Most of the analysis we conducted on the trajectories is independent on the order of the data, for instance, RMSF, PCA, thus randomization is not needed. In the computation of errors in the free energy calculations along a pathway, we used the bootstrap method. Here, data were randomized and a sample of 20% was excluded for each batch.
Blinding	Our in-silico experiments were blinded, starting from a crystal structure, the Newton's equations were solved to capture the dynamics of the protein. These experiments can be fully reproduced and there is not a bias in choosing the data samples.

Reporting for specific materials, systems and methods

We require information from authors about some types of materials, experimental systems and methods used in many studies. Here, indicate whether each material, system or method listed is relevant to your study. If you are not sure if a list item applies to your research, read the appropriate section before selecting a response.

Materials & experimental systems

n/a	Involved in the study	
☒	☐	Antibodies
☒	☐	Eukaryotic cell lines
☒	☐	Palaeontology and archaeology
☒	☐	Animals and other organisms
☒	☐	Human research participants
☒	☐	Clinical data
☒	☐	Dual use research of concern

Methods

n/a	Involved in the study	
☒	☐	ChIP-seq
☒	☐	Flow cytometry
☒	☐	MRI-based neuroimaging
